# Glucocorticoids protect HEI-OC1 cells from tunicamycin-induced cell damage via inhibiting endoplasmic reticulum stress

**DOI:** 10.1515/biol-2021-0057

**Published:** 2021-07-01

**Authors:** Zhibiao Liu, Bing Fei, Lisheng Xie, Jin Liu, Xiaorui Chen, Wenyan Zhu, Lingyun Lv, Wei Ma, Ziwen Gao, Jie Hou, Wandong She

**Affiliations:** Department of Otolaryngology-Head and Neck Surgery, Nanjing Drum Tower Hospital Clinical College of Nanjing Medical University, 321 Zhongshan Road, Nanjing 210008, China; Department of Otorhinolaryngology-Head and Neck Surgery, The Affiliated Huaian No. 1 People’s Hospital of Nanjing Medical University, Huaian, Nanjing, China; Department of Otolaryngology-Head and Neck Surgery, Affiliated Huai’an Hospital of Xuzhou Medical University, 62 South Huaihai Road, Huai’an 223002, China; Department of Otolaryngology-Head and Neck Surgery, Nanjing Drum Tower Hospital, The Affiliated Hospital of Nanjing University Medical School, Jiangsu Provincial Key Medical Discipline, Nanjing, China; Department of Otolaryngology-Head and Neck Surgery, Nanjing Drum Tower Hospital Clinical College of Traditional Chinese and Western Medicine, Nanjing University of Chinese Medicine, Nanjing 210000, China

**Keywords:** endoplasmic reticulum stress, PERK–CHOP pathway, dexamethasone, mifepristone

## Abstract

**Background:**

To analyze mechanisms of action of glucocorticoid treatment for endoplasmic reticulum stress (ERS) in sensorineural hearing loss (SNHL), we aimed to evaluate the expression and activation status of the protein kinase RNA-like ER kinase (PERK)–C/EBP homologous protein (CHOP) pathway, which is the major pathway in the ERS.

**Methods:**

In the present study, we established an *in vitro* ERS model using tunicamycin-treated hair-cell-like HEI-OC1 cells. The effect of dexamethasone on proliferation inhibition, apoptosis, and ATF4–CHOP pathway in HEI-OC1 cells was examined by CCK-8 assay, flow cytometry, western blotting, and reverse transcription PCR, respectively.

**Results:**

In HEI-OC1 cells, dexamethasone was shown to significantly reduce the tunicamycin-induced expression of ATF4 and CHOP in the context of sustained viability and proliferation, a therapeutic effect that was reversible by co-treatment with a glucocorticoid antagonist.

**Conclusion:**

Dexamethasone can protect hair-cell-like HEI-OC1 cells from ERS damage, which may be one of the mechanisms of action for GCs in SNHL treatment.

## 1 Introduction

Endoplasmic reticulum (ER) is an important organelle to maintain normal cellular homeostasis. When eukaryotic cells are exposed to pathophysiological stressors, a large number of misfolded proteins accumulate in the ER and activate endoplasmic reticulum stress (ERS) [1]. ERS is related to many human diseases [1,2]. During the early stages of ERS, cells can adapt to altered environmental conditions by reducing unfolded or misfolded protein. However, if stress conditions persist, cells undergo apoptosis [3]. Protein kinase RNA-like ER kinase (PERK) is a predominant ERS-induced apoptotic signaling pathway and it is activated by phosphorylation, thereby phosphorylating eukaryotic initiation factor 2α (eIF2α). p-eIF2α can promote the expression of activating transcription factor 4 (ATF4) and C/EBP homologous protein (CHOP) [4,5]. After CHOP expression increases considerably, CHOP accumulates in the nucleus and ultimately results in apoptosis [5]. In several animal models of sensorineural hearing loss (SNHL), ERS was believed to be associated with inner ear injuries [6,7,8,9].

Glucocorticoids (GCs) regulate many complex signaling pathways [10,11,12]. It has been reported that, under ERS conditions, there is crosstalk between CHOP and GR signaling, which is associated with a glucocorticoid receptor (GR)-CHOP heterocomplex formation [13].

Therefore, we hypothesized that GCs might protect inner ear cells from ERS damage. In the present study, we examined the effects of GCs on the expression of proteins associated with the PERK–CHOP pathway in HEI-OC1 cells to validate a putative role of ERS in SNHL and to determine whether GCs can reduce ERS.

## 2 Materials and methods

### 2.1 Cell culture and drug administration

HEI-OC1 cells were obtained from the Chinese academy of medical science. The cells were maintained in DMEM medium (Life technologies) supplemented with 10% Fetal Bovine Serum (FBS Life technologies) and 100 U/mL penicillin along with 200 mg/mL streptomycin. 1 × 10^4^ HEI-OC1 cells were seeded in 96-well microplates and cultured for 24 h. Cultures were then assigned to three groups. In the first group, cells were cultured with various concentrations of tunicamycin (TM) (0.1, 0.5, 1, 5, or 10 µg/mL) in DMEM culture medium for 12, 24, 36, or 48 h to determine the optimal concentration and culture time for tunicamycin-mediated inhibition. In the second group, cells were pretreated with various concentrations of dexamethasone (DEX) (0.2, 2, 20, or 200 nmol/mL) for 12 h and then treated with the optimal concentration of tunicamycin in DMEM culture medium to determine the optimal concentration of dexamethasone for reducing tunicamycin-mediated inhibition. In the third group, cells were pretreated with different concentrations of mifepristone (MIF) (0.2, 2, 20, and 200 nmol/mL) and the optimal concentration of dexamethasone for 12 h followed by culturing with tunicamycin to determine the optimal concentration of mifepristone-mediated antagonism of the therapeutic effects elicited by dexamethasone. The inhibition rate of cell proliferation was detected using the CCK-8 Cell Proliferation Detection Kit (Tianjin Bayang Huake Biotechnology Co., Ltd, China.). The optimized conditions were then used to conduct comparative analyses between cultures, using appropriate controls containing no drugs or with individual drug treatments.

### 2.2 Flow cytometry (FACS)

Flow cytometric analysis has been done using Annexin V/Propidium Iodide (PI) Apoptosis Detection Kit (Beyotime, Shanghai, China) according to the manufacturer’s instructions. HEI-OC1 cells (3 × 10^5^) were collected and stained with 5 μL Annexin V-APC and 5 μL PI in the dark at room temperature for 10 min. Data were then acquired on a BD Accuri™ C6 Plus flow cytometer (BD, Franklin Lakes, NJ, USA) and analyzed by Flow Jo V10 software (Tree Star Software, San Carlos, CA, USA).

### 2.3 qPCR and mRNA extraction

Total RNA was extracted from HEI-OC1 Cells using TRIzol reagent. cDNA was then obtained by reverse-transcription. Real-time PCR was performed with the Applied Biosystems QuantStudio 6 Flex Real-Time PCR System (Applied Biosystems, Singapore). The M-MLV was applied to synthesize cDNA through reverse-transcription. For cDNA synthesis, samples were incubated at 43°C for 30 min, 97°C for 5 min, and 5°C for 5 min. The thermal cycle conditions for real-time PCR included an initial denaturation at 95°C for 30 s, followed by 40 cycles of 5 s denaturation at 95°C and 30 s extension at 60°C. Relative fold changes were determined by 2^ΔΔCt^ method [14]. Primer sequences of PERK, eIF2α, ATF4, CHOP, and β-actin that were used in real-time PCR are listed in Table 1.

**Table 1:** Sequences for real-time PCR primers

Gene	Sequences (5′ → 3′)	Amplification efficiency
PERK	F: GTACTGACTCCAATGCCAGCCTA	1.00
	R: CATCTGGGTGCTGAATGGGTA	
eIF2α	F: ATGGTTATGAAGGCATTGATGCTG	1.00
	R: TGTCATCACATACCTGGGTGGAG	
ATF4	F: CTATGGATGATGGCTTGGCCA	1.01
	R:CCAACGTGGTCAAGAGCTCAT	
CHOP	F: AGTGCATCTTCATACACCACCACA	1.02
	R: CAGATCCTCATACCAGGCTTCCA	
β-Actin	F: AGAGGGAAATCGTGCGTGAC	1.03
	R: CAATAGTGATGACCTGGCCGT	

### 2.4 Western blotting

Total protein was extracted from HEI-OC1 Cells by using RIPA buffer with protease and phosphatase inhibitors. Protein concentration was determined by BCA assay. Thirty micrograms of protein were resolved by SDS-PAGE and then transferred onto a PVDF membrane. The membrane was blocked with 5% BSA for 1 h at room temperature and then incubated with the primary antibodies (PERK, or eIF2α, or p-eIF2α, Cell Signaling Tech, USA; p-PERK, ImmunoWay, USA; ATF4, Abcam, UK; CHOP, or BAX, or Bcl-2, Proteintech, USA. 1:1,000/each antibody) at 4°C overnight. After washing with TBST, the membranes were incubated with appropriate secondary antibodies (anti-rabbit IgG, 1:10,000, Fcmacs, China) for 2 h at room temperature. ECL substrate was used to visualize the bands, and the blots were developed by Tanon 5200 Multi fully automatic fluorescence/chemiluminescence image analysis system (Tanon Science & Technology Co, Ltd, Shanghai, China). Protein bands were analyzed for densitometry using NIH Image J software.

### 2.5 Statistical analysis

All data were expressed as mean ± standard deviation. SPSS20.0 software (IBM Corp. Armonk, NY, USA) was used for statistical analyses. The independent samples *t*-test was used to compare the values of means between groups. A value of *p* < 0.05 was considered a statistically significant difference. All experiments have been done in three independent replicas.

## 3 Result

### 3.1 Dexamethasone reversed mifepristone and tunicamycin’s ERS and apoptotic effect in HEI-OC1 cells

**Figure 1:**
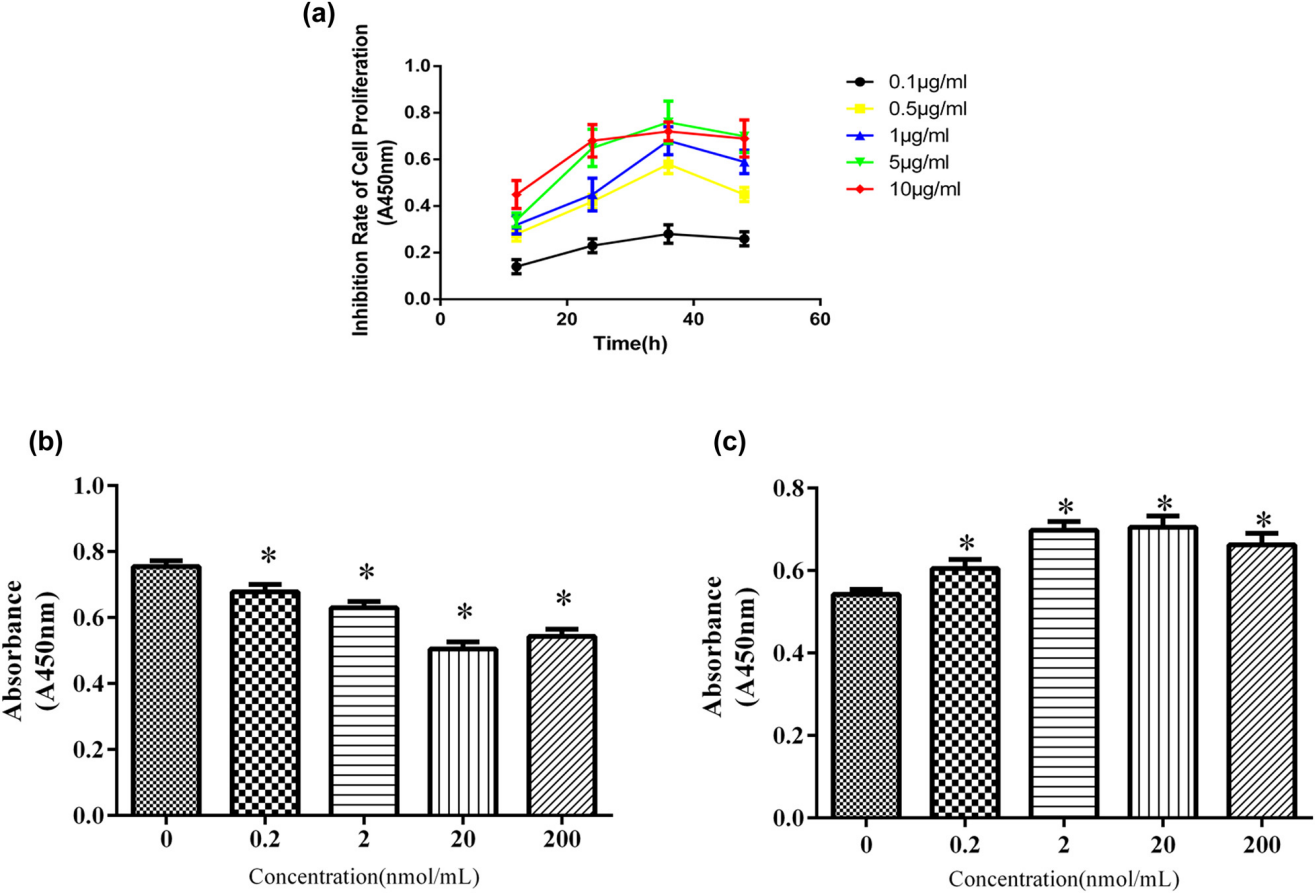
The effects of tunicamycin, dexamethasone, and mifepristone on the proliferation of HEI-OC1 cells. (a) Dose-response of tunicamycin on the proliferation of HEI-OC1 cells. While significant inhibition of HEI-OC1 cell proliferation was observed at all test doses of tunicamycin and at all time points (all *p* < 0.05), the strongest inhibition of tunicamycin on the proliferation of HEI-OC1 cells was observed at 36 h postexposure at a concentration of 5 µg/mL (*p* < 0.05). (b) Dose-response profile of dexamethasone pretreatment on the proliferation of HEI-OC1 cells. HEI-OC1 cells were pretreated with various concentrations of dexamethasone (0–200 nmol/mL) for 12 h before culturing with 5 µg/mL of tunicamycin. Dexamethasone alleviated the inhibition of tunicamycin on HEI-OC1 cell proliferation (all *p* < 0.05). The optimal dose of dexamethasone was 20 nmol/mL (*p* < 0.05). (c) Dose-response profile of mifepristone-mediated antagonism of dexamethasone protection of HEI-OC1 cells from tunicamycin-induced proliferation inhibition. Cells were pretreated with various concentrations of mifepristone (0–200 nmol/mL) and 20 nmol/mL of dexamethasone for 12 h and then cultured with 5 µg/mL tunicamycin for 36 h. Mifepristone reversed the protective effect of dexamethasone (all *p* < 0.05). The optimal concentration of mifepristone was 20 nmol/mL (*p* < 0.05). All results are expressed as \bar{X}\pm \text{SD}], * indicates *p* < 0.05.

HEI-OC1 cells were treated with various concentrations (0.1, 0.5, 1, 5, 10 µg/mL) of tunicamycin (TM) for 12, 24, 36, and 48 h. TM inhibited the proliferation of HEI-OC1 cells in a dose- and time-dependent manner. TM (5 µg/mL – 36 h) significantly inhibit the proliferation of HEI-OC1 cell in both time and dose-dependent manners (*p* < 0.05) (Figure 1a). HEI-OC1 cells were then pretreated with various concentrations (0.2, 2, 20, or 200 nmol/mL) of glucocorticoid dexamethasone (DXM); after 12 h, 5 µg/mL of TM was added into the medium and cells were cultured for additional 36 h. The optimal concentration of DXM was found to be 20 nmol/mL (Figure 1b). All doses of DXM reduced the TM-induced inhibition of HEI-OC1 cell proliferation (all *p* < 0.05).

To understand the glucocorticoid receptor (GR) role in this therapeutic response, HEI-OC1 cells were pretreated with the optimal therapeutic dose of DXM(20 nmol/mL) in the presence of various concentrations (0.2, 2, 20, or 200 nmol/mL) of the GR antagonist, mifepristone (MIF). After 12 h, 5 µg/mL of TM was added into the medium, and the cells were cultured for an additional 36 h. All test doses of MIF reduced the protective effect of dexamethasone (20 nmol/mL) against TM-induced inhibition of HEI-OC1 cell proliferation (*p* < 0.05, 1C).

**Figure 2:**
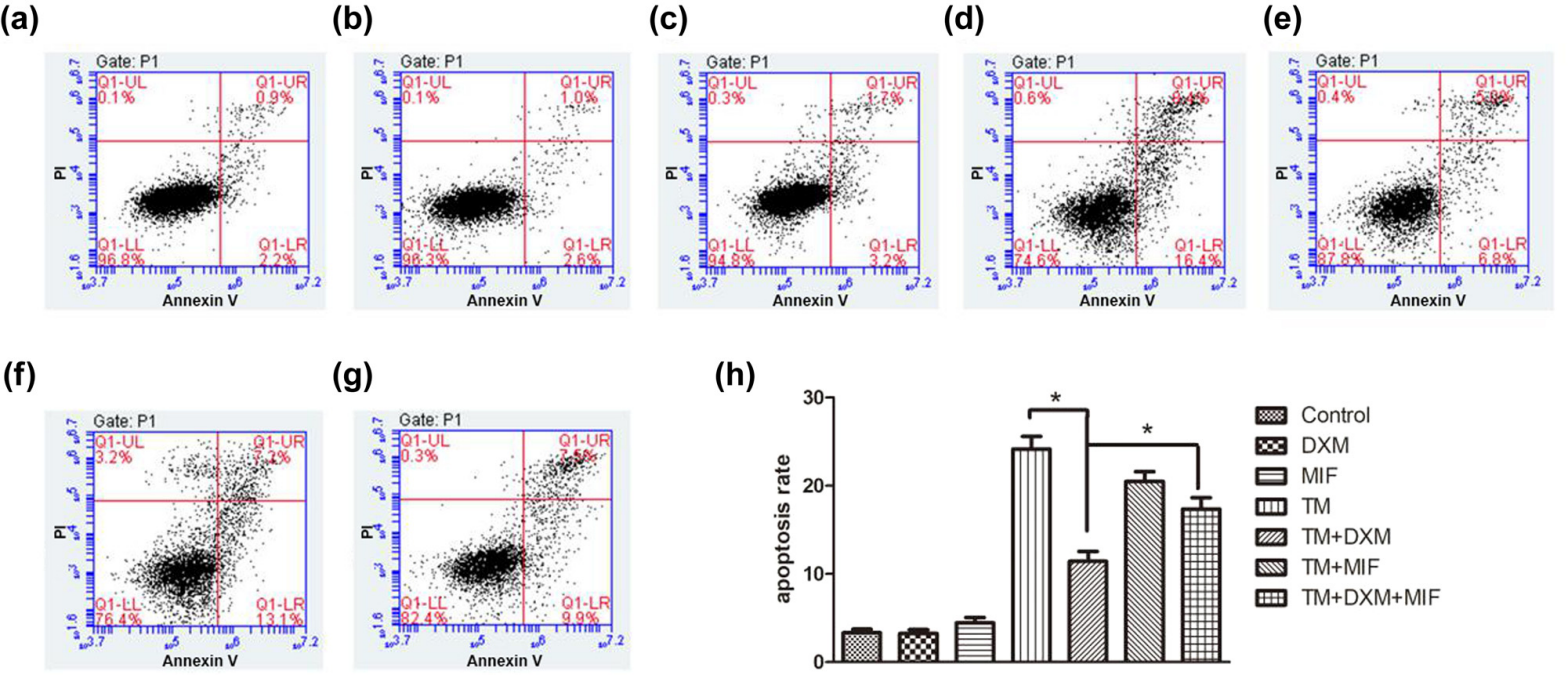
Dexamethasone protects HEI-OC1 cells from tunicamycin-induced apoptosis. Examples of flow cytometry analysis of apoptosis in the control group (a), the DXM group (b), the MIF group (c), the TM group (d), the TM + DXM group (e), the TM + MIF group (f), and the TM + DXM + MIF group (g). Apoptosis rates were statistically analyzed (h). Compared to the control group, no increased apoptosis was observed in the DXM and MIF groups (all *p* > 0.05). Significantly more apoptosis was observed in the TM, TM + MIF, TM + DXM, and TM + DXM + MIF groups compared to the control group (all *p* < 0.05). However, dexamethasone treatment (TM + DXM) significantly protected HEI-OC1 cells from tunicamycin-induced apoptosis, and mifepristone (TM + DXM + MIF) reversed this protective effect (all *p* < 0.05). Mifepristone pretreatment did not alter the apoptosis rate in the TM + MIF group compared to the TM alone group (*p* > 0.05). All results are expressed as \bar{X}\pm \text{SD}], * indicates *p* < 0.05.

Investigating DXM potential protection against the damage induced by ERS, HEI-OC1 cells were incubated with 5 µg/mL of TM alone or following pretreatment with DXM (20 nmol/mL), MIF (20 nmol/mL), or DXM + MIF. Flow cytometry was used to detect apoptosis. Compared to the normal control group, no increased apoptosis was observed in cells treated with DXM or MIF alone (*p* > 0.05). Increased apoptosis was observed in the TM, TM + DXM, TM + MIF, and TM + DXM + MIF groups compared to the control group (*p* < 0.05, Figure 2d–g). However, apoptosis was significantly decreased in the TM + DXM group compared to the TM group (*p* < 0.05, Figure 2d and e), and more apoptotic cells were counted in the TM + DXM + MIF group compared to the TM + DXM group (*p* < 0.05, Figure 2e and g). These results indicate that TM-induced ERS promoted apoptosis in HEI-OC1 cells and that DXM-mediated protection of HEI-OC1 cells from this pathological response could be reversed by MIF.

### 3.2 Tunicamycin upregulated the expression of ATF-4 and CHOP proteins in HEI-OC1 cells

**Figure 3:**
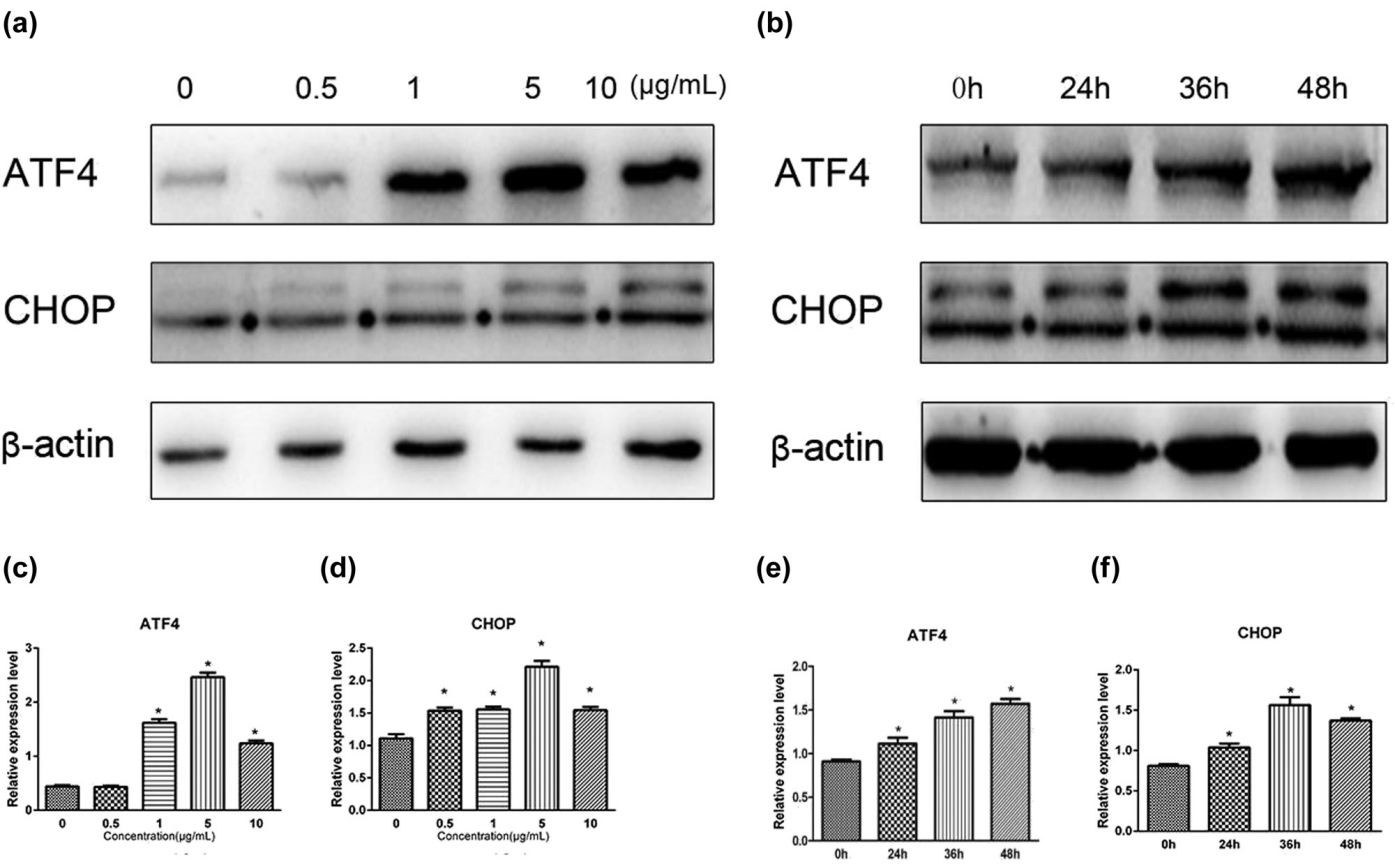
Effects of tunicamycin on the expression of ATF4 and CHOP in HEI-OC1 cells. (a) Example of western blots of ATF4 and CHOP protein expression in HEI-OC1 cells treated with various concentrations of tunicamycin (0–10 µg/mL) for 36 h. The expression of ATF4 and CHOP proteins in HEI-OC1 cells gradually increased with increasing concentrations of tunicamycin. (b) Example of western blots of ATF4 and CHOP protein expression in HEI-OC1 cells cultured with 5 µg/mL of tunicamycin for various culture times (0–48 h). The expression of ATF4 and CHOP proteins in HEI-OC1 cells gradually increased in the presence of TM with longer culture times. The western blots were quantitatively analyzed (c–f). A dose-response on ATF4 and CHOP expression was observed across the concentration range of 0.5–5 µg/mL of tunicamycin (all *p* < 0.05, c and d). At a fixed dose of 5 µg/mL tunicamycin, increased ATF4 protein was expressed when the cells cultured longer (all *p* < 0.05, e). Similar time-dependent effects on protein expression were also observed for CHOP when the cells were cultured for 24–36 h in the presence of tunicamycin (all *p* < 0.05, f). All results are expressed as \bar{X}\pm \text{SD}], * indicates *p* < 0.05.

To study the effects of TM-induced ERS at the molecular level, HEI-OC1 cells were treated with increasing concentrations of TM (0–10 µg/mL) between 0 and 48 h. The expression of ATF4 and CHOP proteins was significantly increased with increasing TM concentrations and culture times (Figure 3a and b). A dose-response profile for ATF4 and CHOP expression was observed across the 0.5–5 µg/mL concentration range of TM (*p* < 0.05, Figure 3c and d). This TM-induced ATF4 and CHOP expression were significantly increased with extended cultured intervals (*p* < 0.05, Figure 3e and f).

### 3.3 Effects of dexamethasone and mifepristone on the upregulation of ATF-4 and CHOP induced by tunicamycin in HEI-OC1 cells

**Figure 4:**
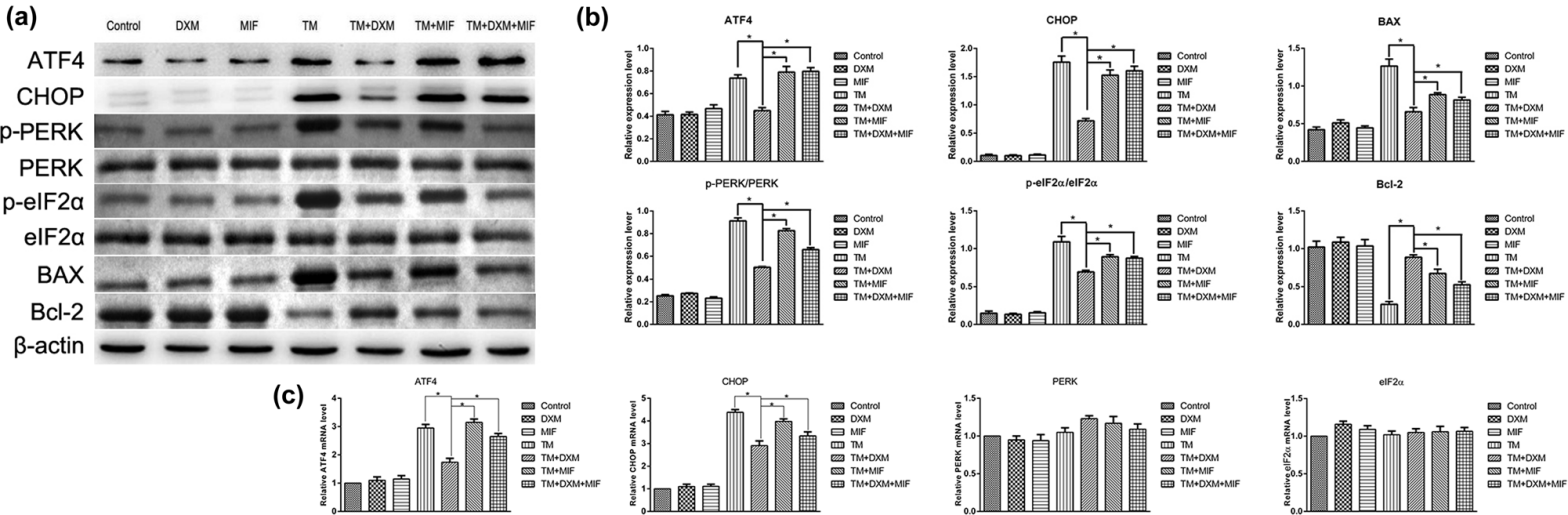
Effects of dexamethasone and mifepristone on the upregulation of ATF-4 and CHOP induced by tunicamycin in HEI-OC1 cells. (a and b) Example of western blots of ATF4, CHOP, PERK, eIF2α, BAX, and Bcl-2 expression after drug treatment. Upregulation of ATF4, CHOP, BAX, PERK, and eIF2α expression and downregulation of Bcl-2 were observed in the TM, TM + MIF, and TM + DXM + MIF groups. The upregulation of ATF4, CHOP, BAX, PERK, and eIF2α and the downregulation of Bcl-2 were blocked by dexamethasone in the TM + DXM group. (c) Similar results were also observed in the expression pattern of ATF4 and CHOP mRNA levels in each test group. The expression of PERK and eIF2ɑ mRNA was not changed after drug treatment (all *p* > 0.05). All results are expressed as \bar{X}\pm \text{SD}], * indicates *p* < 0.05.

The ERS-related expression of PERK, eIF2α, ATF4, and CHOP in HEI-OC1 cells was then examined by western blot and Qrt-PCR in the context of therapeutic pretreatment with DXM. TM treatment significantly upregulated the protein expression of BAX, p-PERK, p-eIF2α, ATF4, and CHOP (Figure 4a and b), downregulated the protein expression of Bcl-2, and upregulated mRNA expression of ATF4 and CHOP (*p* < 0.05) (Figure 4c); pretreatment with DXM reversed TM’s effect (all *p* < 0.05, Figure 4a and b). DXM-mediated inhibition of ERS was reversed by co-treatment with MIF, demonstrating specificity for the antagonistic response at the molecular levels. These results suggest that DXM protects HEI-OC1 cells from ERS-induced apoptosis by inhibiting BAX, p-PERK, p-eIF2α, ATF4, and CHOP expression and increasing the Bcl-2 expression.

## 4 Discussion

GCs have vast effects on the metabolic, immunological, and homeostatic functions. In the inner ear, it directly targets the glucocorticoid receptor (GR) [15]. After GCs were delivered in the inner ear, thousands of inner ear genes were affected and this number increased significantly [16]. GCs have been widely used in the protection of inner ear injury. For example, GCs could significantly improve the auditory brainstem response threshold after acoustic overexposure [17]. Also, recently many hospitals consider GCs being applied perioperatively in patients undergoing cochlear implantation as a promising treatment regimen [18]. On the other hand, high doses of corticosterone can impair auditory nerve processing [19].

ERS is considered a common cause of various sensorineural deafness. Sensorineural hearing loss is reported to be associated with ERS in animal studies. Whether GCs can protect inner ear cells by inhibiting ER stress remains unclear [20,21,22]. We speculate that ERS may be prominent in the inner ear cells of patients suffering from SNHL. However, the relationship between GCs and ERS is immensely complicated, and the effects of GCs whether inhibiting or promoting ERS can differ upon different cells [23,24]. Question remains, if the inner ear cells are damaged in SSNHL patients, will GCs promote or inhibit ERS? To answer this question, we used tunicamycin (the most common drug used to induce ERS) to treat hair-cell-like HEI-OC1 cells as an *in vitro* system for modeling inner ear ERS damage. Additionally, we pretreated HEI-OC1 cells with GCs to investigate whether GCs have protective effects against ERS damage. We found that dexamethasone can effectively protect HEI-OC1 cells from ERS damage. Also, these effects could be inhibited by mifepristone, a well-studied GC antagonist.

In order to explore the relationship between ERS and apoptosis, we further determined the protein expression of BAX and Bcl-2 in HEI-OC1 cells. We found that TM significantly upregulated the protein expression of BAX and downregulated the protein expression of Bcl-2 in HEI-OC1. Previous studies have found that ERS can induce apoptosis in H9c2 cell and MLTC-1 cells, which was similar to our results. To determine the role of GCs in ERS-induced apoptosis, we examined the protein expression of ERS marker genes. Interestingly, GCs not only inhibited the expression of p-PERK, p-eIF2α, ATF4, and CHOP, but also reversed the expression of apoptosis-related proteins. These results indicated that GCs may reduce apoptosis by alleviating ERS.

**Figure 5:**
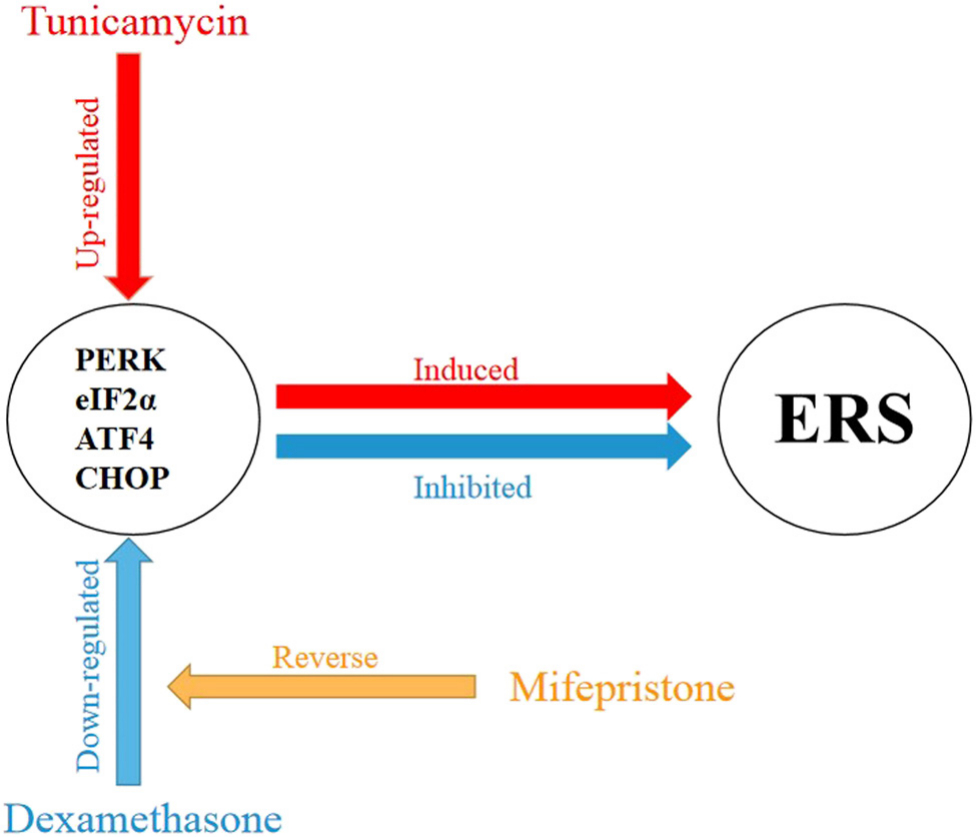
Mechanisms of action of glucocorticoid treatment ERS in SNHL.

After binding to GRs, GCs enter the nucleus and control the activity of large gene networks associated with a variety of developmental and metabolic processes [25]. GCs may inhibit ERS and protect cells through multiple pathways. For instance, GCs can inhibit ERS by promoting the secretion of correctly folded proteins and degradation of misfolded proteins; GRs may undergo re-localization and phosphorylation by ERS inducers, thereby decreasing ERS; GCs can alleviate ERS response by inducing leucine zippers, and the interactions of GR-bound GCs with CHOP can reverse tunicamycin-induced cell death [13,24,26]. To determine the molecular mechanisms of GC-mediated mitigation of ERS damage in inner ear cells, dexamethasone and mifepristone were used to pretreat HEI-OC1 cells in this study. The Figure 5 was drew to explain the mechanisms of action of glucocorticoid treatment ERS in SNHL.

We found that dexamethasone could suppress tunicamycin-induced increases in ATF4 and CHOP expression in HEI-OC1 cells. Attenuation of ERS in inner ear cells may, therefore, represent an important mechanism of action for GCs to elicit their therapeutic effects in patients with SNHL.

In conclusion, our results suggest that GCs can inhibit ERS-related ATF4 and CHOP expression and confer protective effects against ERS damage and potential apoptosis in inner ear cells; and also that GCs may alleviate SNHL by inhibiting ERS, which may be one of the mechanisms of action for GC treatment in patients with SNHL. This study provided a theoretical basis for clinical treatment of SNHL.
